# Comparative Analysis of Metabolite Profiling of *Momordica charantia* Leaf and the Anti-Obesity Effect through Regulating Lipid Metabolism

**DOI:** 10.3390/ijerph18115584

**Published:** 2021-05-24

**Authors:** Meiqi Fan, Jae-In Lee, Young-Bae Ryu, Young-Jin Choi, Yujiao Tang, Mirae Oh, Sang-Ho Moon, Bokyung Lee, Eun-Kyung Kim

**Affiliations:** 1 Division of Food Bioscience, College of Biomedical and Health Sciences, Konkuk University, Chungju 27478, Korea; fanmeiqi@kku.ac.kr (M.F.); moon0204@kku.ac.kr (S.-H.M.); 2 Natural Product Material Research Center, Korea Research Institute of Bioscience and Biotechnology, Jeongeup 56212, Korea; Lji613@kribb.re.kr (J.-I.L.); ybryu@kribb.re.kr (Y.-B.R.); 3 Department of Food Science and Nutrition, College of Health Science, Dong-A University, Busan 49315, Korea; choijang11@kku.ac.kr (Y.-J.C.); bolee@dau.ac.kr (B.L.); 4 Center for Silver-Targeted Biomaterials, Brain Busan 21 Plus Program, Dong-A University, Busan 49315, Korea; 5 School of Bio-Science and Food Engineering, Changchun University of Science and Technology, Changchun 130600, China; yuanxi00@126.com; 6 Grassland and Forages Division, National Institute of Animal Science, Rural Development Administration, Cheonan 31000, Korea; miraeoh@korea.kr

**Keywords:** *Momordica charantia* leaf, metabolite profiling, obesity, lipid metabolism

## Abstract

This study investigated the effects of *Momordica charantia* (*M. charantia*) extract in obesity and abnormal lipid metabolism in mice fed high fat diet (HFD). Fruit, root, stem, and leaf extracts of *M. charantia* were obtained using distilled water, 70% ethanol and 95% hexane. *M. charantia* leaf distilled water extract (MCLW) showed the highest antioxidant activity in both 2,2-diphenyl-1-picrylhydrazyl (DPPH) radical scavenging activity tests and reducing power. Metabolite profiles of *M. charantia* leaf extracts were analyzed for identification of bioactive compounds. HFD-fed mice were treated with MCLW (oral dose of 200 mg/kg/d) for 4 weeks. MCLW reduced lipid accumulation, body weight, organ weight, and adipose tissue volume and significantly improved glucose tolerance and insulin resistance in HFD mice. Furthermore, MCLW administration reduced serum total cholesterol and low-density lipoprotein cholesterol, and increased serum high-density lipoprotein cholesterol compared with HFD mice. Moreover, MCLW significantly reduced the levels of serum urea nitrogen, alanine aminotransferase, alkaline phosphatase, and aspartate aminotransferase; alleviated liver and kidney injury. MCLW decreases expression of genes that fatty acid synthesis; increase the expression of catabolic-related genes. These results indicate that MCLW has an inhibitory effect on obese induced by high fat diet intake, and the mechanism may be related to the regulation of abnormal lipid metabolism in liver and adipose tissue, suggesting that MCLW may be a suitable candidate for the treatment of obesity.

## 1. Introduction

Obesity is a chronic metabolic disease with global prevalence, which is mainly characterized by the abnormal distribution or excessive accumulation of fat caused by long-term excess energy in the body [1]. Adverse lifestyle, genetic, and environmental factors are all involved in the pathogenesis of obesity [2]. Obesity can induce systemic energy metabolism disorder and increase the risk of cardiovascular diseases, digestive diseases, respiratory diseases, endocrine diseases, and psychological diseases [3]. Studies on the pathogenesis of metabolic syndrome caused by obesity have found that the activation of adenylate-activated protein kinase K (AMPK) drives the activation of downstream acetyl-CoA carboxylase (ACC); it also inhibits cholesterol response element-binding protein 1c (SREBP1c), fatty acids Synthetase (FAS) transcription and expression, thereby inhibiting the synthesis of fatty acids and cholesterol [4]. Therefore, AMPK and its downstream signaling pathways have become a new target for the treatment of obesity.

With the development of anti-obesity drugs, some drugs have been approved and marketed. However, the vast majority of drugs were withdrawn due to their severe side effects [5]. Orlistat is a powerful and particular intestinal lipase inhibitor, it is now the most commonly used as an anti-obesity drug, but its use results in several adverse intestinal reactions, such as oily feces, diarrhea, abdominal pain, and fecal spots [6]. Considering these adverse effects, using natural products as raw materials to develop safe, efficient and inexpensive obesity treatments is one of the hot research topics at present. Since ancient times, plants and their extracts have received immense attention because of their safety and anti-obesity effects [7].

*Momordica charantia* (bitter gourd) fruit is rich in polysaccharides, saponins, and polyphenols, and contains peptides, which have long been used to prevent and alleviate diabetes. Animal experiments have confirmed that *M. charantia* fruit has the effects of improving insulin resistance and regulating glucose and lipid metabolism [8]. Various parts of *M. charantia*, such as stems, leaves, and roots contain many biologically active compounds, such as glycosides, phytosterols, alkaloids, phenolic, saponins, and flavonoids [9,10,11], and have been used as a traditional medicine for curing ailments such as toothache, indigestion, and diabetes [12,13]. *M. charantia* tea made from dried slices, called herbal tea, has medicinal applications [14], and it was observed that consumption of this tea resulted in reduced body fat accumulation and weight gain while the cellular glucose uptake was increased [15]. As a natural food, the complex composition of *M. charantia* will inevitably lead to the diversity of its effects. At present, studies on *M. charantia* mainly focus on its effect on diabetes, and there have been only a few studies on fat deposition; the precise mechanisms are still to be explained.

In this research, distilled water, 70% ethanol, and 95% hexane were used for extraction solvent for *M. charantia* fruits (MCF), roots (MCR), stems (MCS), and leaves (MCL). Following the antioxidant and preadipocyte differentiation experiments were performed in order to select one of the extracts for the in vivo experiments. In addition, a comparative analysis of metabolite profiling of MCL distilled water (MCLW), ethanol (MCLE), and hexane extract (MCLH) was conducted to investigate the active components of each extract. A HFD-induced obese mouse model was used to assess anti-obesity activity of MCLW. Finally, the effects of MCLW on the expression of key genes involved in obesity and lipid metabolism were evaluated.

## 2. Materials and Methods

### 2.1. Extraction and Preparation of Sample

**Figure 1:**
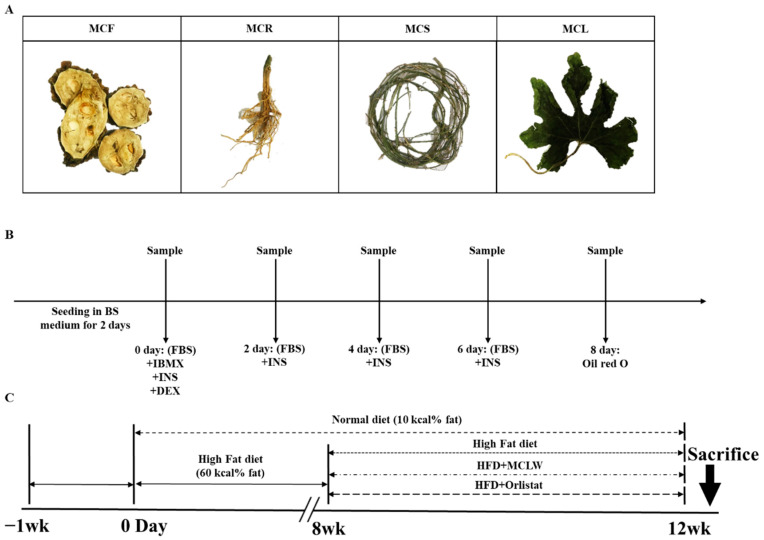
Background experimental information. Four parts of *M. charantia* used in antioxidant experiments, viz., fruit (MCF), root (MCR), shoot (MCS), and leaf (MCL) (**A**). Design of 3T3-L1 cell differentiation experiments (**B**). Timeline for the in vivo study (**C**). BS: bovine serum; FBS: heat-inactivated fetal bovine serum; IBMX: 3-Isobutyl-1-methylxanthine; INS: insulin; DEX: Dexamethasone; HFD: High fat diet.

Fresh *M. charantia* fruit, root, stem, and leaf (Shandong, China, Figure 1A) were air-dried in shade at room temperature (RT) for 2 weeks, followed by crushing the sample to powder. The powdered samples were stored at room temperature before extraction. Solvents (distilled water, 70% ethanol, and 95% hexane) were employed for the extraction. For the extraction efficiency, 100 g powder of fruit, root, stem, and leaf were separately soaked in 2 L canonical flasks containing 1 L solvent (three different flasks with different solvent for each plant part). The flasks, containing soaked plant powder, were sealed with a cotton plug and aluminum foil and placed on a shaker at RT for 1 h (distilled water), 2 h (70% ethanol), and 6 h (95% hexane). The process was repeated thrice to ensure maximum extraction. The pooled extracts were then concentrated by using a rotary evaporator at 60 °C, followed by filtration, and lyophilization for 5 days.

### 2.2. Antioxidant Activity Analysis

2,2-diphenyl-1-picrylhydrazyl (DPPH) radical scavenging activity, and reducing power were performed to measure the in vitro antioxidant activities of the 12 different extracts from *M. charantia*, following the procedure reported by Blois and Oyaizu [16,17].

### 2.3. UPLC-Q-TOF MS Investigation of the Fragmentation Patterns

The Vion UPLC™ system (Vion, Waters, Milford, MA, USA) was used and LC conditions were optimized within 9 min on an Acquity UPLC BEH C18 column (2.1 × 100 mm, 1.7 μm; Waters, Milford, MA, USA) and the temperature was set at 55 °C. The flow rate was set at 0.35 mL/min. The mobile phases included (**A**) water with 0.1% formic acid (FA) and (**B**) acetonitrile (ACN) with 0.1% FA. Spectrometer was used with electrospray ionization (ESI) in negative modes. The MS operating conditions were as follows: capillary voltage 2.5 kV, sample cone 20 V, ion source temperature 200 °C, desolvation temperature 400 °C, cone gas 30 L/h, desolvation gas 900 L/h, scan time 0.2 s, scan range *m*/*z* 50–1500, and collision energy ramp from 10–30 eV (*m*/*z* 50–1000). 

MarkerLynx software (Waters, Milford, MA, USA) was used to collect, normalize, and align the MS data sets analyzed by UPLC-Q-TOF MS (Waters, Milford, MA, USA). Peaks were collected using a peak-to-peak baseline noise of 1, noise elimination of 6, peak-width at 5% height of 1 s, and an intensity threshold of 10,000. The MS data were aligned with a 0.05 Da mass window and a retention time window of 0.2 min. Both mass spectra were standardized with an internal standard (terfenadine [M + H] and zidovudine [M − H]). The Human Metabolome Database (HMDB) (www.hmdb.ca) (accessed on 23 September 2020), METLIN database (metlin.scripps.edu) (accessed on 23 September 2020), Chemspider (www.chemspider.com) (accessed on 23 September 2020), literature references, and authentic criteria were used to identify the metabolites. This analysis of the fragmentation patterns was conducted three times. All of data showed repeatability, and the representative chromatography was shown.

### 2.4. Cell Culture, Differentiation, and Treatments

The 3T3-L1 cells obtained from the Korean Cell Line Bank (Seoul, Korea) were cultured and maintained in DMEM medium with 10% heat-inactivated fetal bovine serum (FBS) and 1% penicillin/streptomycin (P/S) at 37 °C with a humidified atmosphere of 5% CO_2_. To induce differentiation, two-day post-confluent preadipocytes (designated day 0) were cultured in a hormone mixture differentiation medium, 0.5 mM IBMX, 1 μM dexamethasone, and 5 μg/mL insulin for 2 days. The cells were then cultured for another 2 days in DMEM containing 1% P/S, 10% FBS, and 5 μg/mL insulin. Thereafter, the culture media was replaced with DMEM containing 1% P/S, 10% FBS for 2 days, and then replaced with the medium every 2 days until Day 8 when the adipocytes had acquired intracellular lipid droplets [18]. MCLW (1 mg/mL) and *M. charantia* leaf ethanol extract (MCLE) (1 mg/mL) was administered four times every two days from day 0 to day 6. Control group cells comprised identical media composition without the *M. charantia* extract. The influence of the extract on fat deposition was explained by the size of lipid droplets differentiated from 3T3-L1 preadipocyte.

### 2.5. Determination of Lipid Accumulation by Oil-Red O Staining

3T3-L1 cells, as described in Section 2.4, were incubated on a 12-well plate to induce adipocyte differentiation for 8 days. The Oil-Red O cell lipid index was obtained by rinsing the cells with normal saline on the eighth day, followed by fixing with 10% formaldehyde solution, and staining with Oil Red O solution (0.7 g in 200 mL isopropyl alcohol) at RT for 60 min. Following staining of lipid droplets, the staining solution was removed by rinsing with distilled water, followed by drying. Lipid droplets of 3T3-L1 adipocytes were observed under the microscope. Finally, the stain retained in the cells was eluted with isopropanol, and absorbance was measured at 520 nm using a microplate reader. 

### 2.6. Experimental Animals, Diet, and Treatments

Four-week-old C57BL/6 male mice were purchased from the Nara Bio animal center (NARA Biotech, Seoul, Korea), acclimatized for 1 week at the experimental facility, and housed in groups of four inside transparent plastic cages with aspen chips, under clear pathogen-free conditions, and provided with standard mouse chow diet and tap water ad libitum when not treated. The animal environment was adequately controlled, with a dark-light period of 12 h at 20–21 °C, and relative humidity of 40–45%. All experiments were approved by the Konkuk University Institutional Animal Care and Use Committee, and every possible effort was made to minimize the suffering and the number of animals used in this research (KU17089). 

After 1 week of acclimatization, mice were randomly divided into two groups: mice fed a normal diet (CON, 10% fat, 70% carbohydrate, 20% protein, total 3.85 kcal/g; D12450B, Research Diets Inc., New Brunswick, NJ, USA, *n* = 10), and mice fed with high-fat diet (HFD, 60% fat, 20% carbohydrate, 20% protein, total 5.24 kcal/g; D12492, Research Diets Inc., New Brunswick, NJ, USA, *n* = 30). Food intake of the mice was recorded daily, and their body weights were assessed once a week. After 8 weeks, mice with body weight 20% higher than the CON group were selected, and randomly divided into three groups (*n* = 8) for treatments, 0.9% saline (MCLW vehicle)-treated HFD-fed mice, MCLW (HFD + MCLW; oral dose of 200 mg/kg/d), and orlistat (HFD + orlistat; oral dose of 60 mg/kg/d), for four weeks. Also, CON group (*n* = 8) mice switched to a normal diet and treated with vehicle for four weeks. Orlistat has been used as a positive control. The mice were subjected to overnight fasting after 12 weeks and then sacrificed (Figure 1C).

Blood samples were collected for further analysis. Adipose tissue (epididymis, subcutaneous, visceral, and scapular) was weighed, epididymal fat and liver was collected, frozen in liquid nitrogen, and preserved at −80 °C for further examination. For subsequent histological analyses, sections of epididymal fat and liver tissue were fixed with 10% formaldehyde.

### 2.7. Biochemical Analysis

The cardiac puncture was used to collect blood samples, and serum was separated by centrifugation (3000 rpm for 20 min) and stored at −80 °C until further analysis. An automated analyzer (Abaxis VETSVAN VS2 Chemistry Analyzer, Union City, CA, USA) was used to test serum levels of blood urea nitrogen (BUN), alanine aminotransferase (ALT), alkaline phosphatase (ALP), aspartate aminotransferase (AST), and serum albumin (ALB). A rapid blood lipid analyzer (OSANG healthcare Lipid Pro, Anyang, Republic of Korea) was used to measure serum total cholesterol (TC), low-density lipoprotein cholesterol (LDL), high-density lipoprotein cholesterol (HDL), and triglycerides (TG). Serum leptin was quantified using an ELISA leptin kit (Merck, Darmstadt, Germany).

### 2.8. Histological Analysis

After blood collection, dual-energy X-ray absorptiometry (DXA) measurements were performed with a total-body scanner (InAlyzer dual X-ray absorptiometry, Medikors, Gyeonggi, Korea). The liver was divided into two pieces for paraffin and frozen sectioning. The liver and epididymal adipose tissue were dissected for histological examination, buffered in 10% neutral formalin, and then embedded in paraffin. With paraffin sections (4 μm), hematoxylin and eosin (H&E) staining were conducted. Samples were imaged at 200× under a microscope. With frozen sections (10 μm with OCT compound), lipid accumulation was evaluated with Oil Red O staining. The slices were microscopically imaged at 200×, and the size of fat cells was recorded.

### 2.9. MRNA Expression Analysis

Total RNA was collected from mice livers, and epididymal adipose tissues, and qPCR was conducted, as described previously [19]. PCR reactions were carried out using AccuPower^®^ PCR PreMix (Bioneer, Chungwon, Korea) and a Veriti™ 96-Well Thermal Cycler (Applied Biosystems, Foster City, CA, USA). The primers used in this study were synthesized by Enotech Co. (Daejeon, Korea) (Table S1). GAPDH was chosen in qPCR as an internal references, and the relative quantification of gene expression was determined with the 2^−∆∆Ct^ method.

### 2.10. Protein Expression Analysis

Frozen mice livers and epididymal adipose tissues were homogenized in liquid nitrogen. For Western blot analysis, tissues were lysed in RIPA lysis buffer containing 1% PI. The cell lysates were incubated on ice for 1 h and the homogenates were centrifuged at 4 °C for 20 min at 13,000 rpm. Western blotting was carried out as described previously [20].

### 2.11. Statistical Analysis

The statistical data evaluations were expressed as mean ± SEM. The data was analyzed using SPSS version 11.5 (SPSS Inc., Chicago, IL, USA). Multigroup comparisons of the means using one-way analysis of variance (ANOVA) followed by Dunnett’s multiple comparison post hoc analyses. Otherwise, Student’s *t*-test was performed. For each behavioral test, the false discovery rate (FDR) was determined using the Benjamini–Hochberg procedure with a critical value for false discovery of 0.05. In all cases, *p* < 0.05 values were deemed statistically significant.

## 3. Results

### 3.1. Effects of M. charantia on Antioxidant Activity and Lipid Accumulation

**Figure 2:**
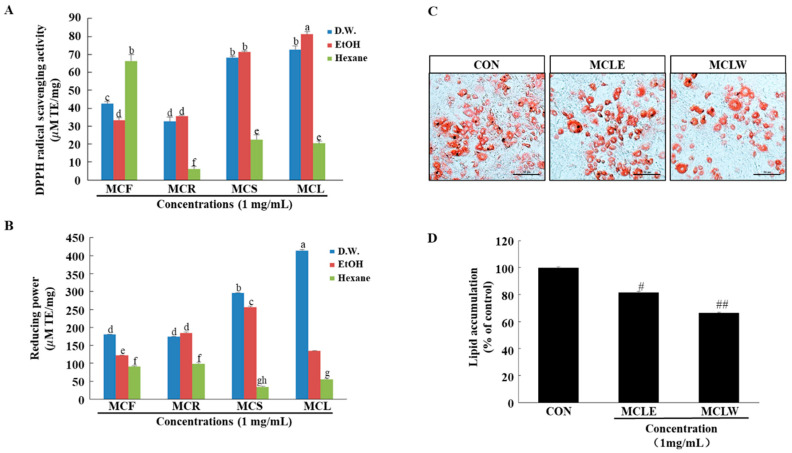
Effect of *M. charantia* on antioxidant and anti-obesity activity. DPPH radical scavenging activity (**A**). Reducing power (**B**). Lipid accumulation was measured using Oil-Red O staining. Sample-treated 3T3-L1 adipocytes (**C**), and lipid levels in 3T3-L1 adipocytes (**D**). Original magnification: 200×. ^a–h^ Values with different superscripts are significantly different at *p* < 0.05 as analyzed by Dunnett’s multiple range tests. Data are mean ± SEM. *n* = 3 per group, ^#^ *p* < 0.05, ^##^ *p* < 0.01 vs. the control (CON).

Obesity is associated with excessive oxidative stress [21,22,23,24,25,26]. Therefore, we investigated the antioxidant activity of different extracts from *M. charantia*. Reducing power assays and DPPH radical scavenging activity were used to evaluate the antioxidant activities of the different extracts. As presented in Figure 2A,B, the antioxidant activity of MCLW and MCLE were significantly higher than the other extracts. Thus, the cytotoxicity of MCLW and MCLE on the preadipocyte 3T3-L1 was examined. We found that MCLW and MCLE had no cytotoxic effect on 3T3-L1 (Figure S1). Hence, these extracts were selected for further assessments.

To examine the antiadipogenic potential of the MCLW and MCLE on the differentiation of preadipocytes into adipocytes, 3T3-LI preadipocytes were treated with MCLW and MCLE for 8 days (from Days 0 to 8). Microscopic inspection of MCLW and MCLE treated cells cultured under optimal differentiation conditions revealed a significant reduction in the accumulation of intracellular lipids. MCLW and MCLE’s inhibitory effects on adipogenesis in 3T3-L1 adipocytes were measured by Oil-Red O staining (Figure 2C). On Day 8 post induction, oil droplets were seen in 3T3-L1 cells, cells were highly stained with the lipophilic Oil-Red O to demonstrate triglyceride accumulation (Figure 2C). In contrast, the degree of staining with Oil-Red O decreased with treatment of MCLW or MCLE, indicating that MCLW or MCLE reduced lipid accumulation (Figure 2C,D). Among the tested MCLW and MCLE samples, MCLW exhibited the highest antiadipogenic potential. These findings suggest that MCLW blocks the differentiation of 3T3-L1 cells to adipocyte. Thus, it was used in subsequent experiments in vivo.

### 3.2. Identification of the Bioactive Components in M. charantia

**Figure 3:**
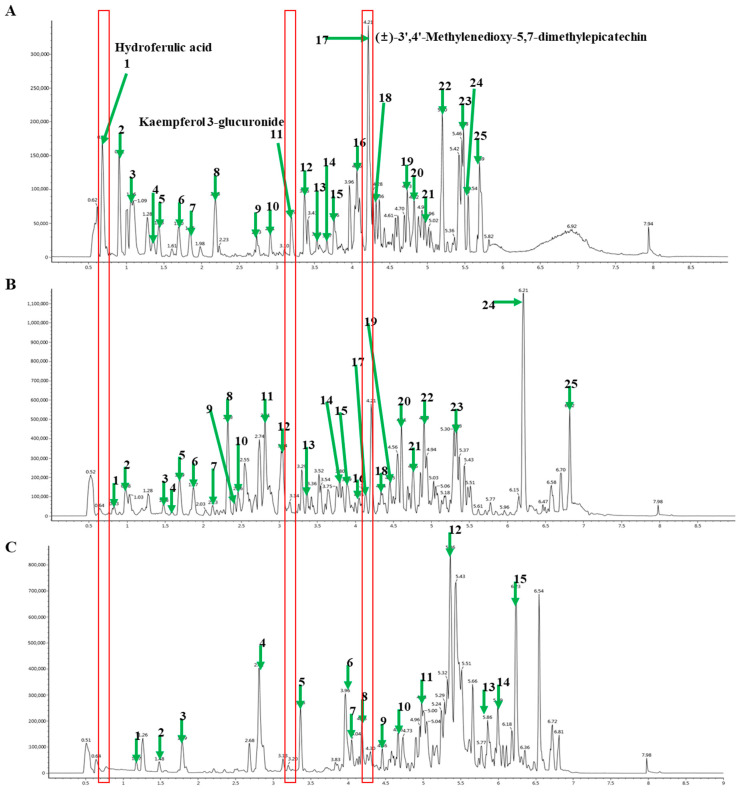
Representative ultra-performance liquid chromatography-quadrupole-time-of-flight mass spectrometry (UPLC-Q-TOF MS) profiles of *M. charantia* leaf. (**A**), D.W extract; (**B**), EtOH extract; and (**C**), hexane extract analyzed by ESI-negative mode (numbering in every panel is independent).

The chemical components of different extracts of *M. charantia* were analyzed using UPLC-Q-TOF MS. Extracts were used to compare the ion flow chromatogram. The retention time and major mass spectrum signals of each extract were analyzed and the major chemical components in *M. charantia* were determined. The number and shape of the chromatographic peaks of the extracts obtained by the different extraction methods varied (Figure 3A–C). The retention time and mass spectrum signal of reference substances were compared in MCLW (Figure 3A), and the retention time and mass spectrum letter of each chromatographic peak were analyzed, compounds in all extracts were identified, mainly flavonoids, triterpenes, and phenolic acids, as presented in Table 1. The test results show that with the increasing polarity of the crude extract, the total flavonoids as well as the degree of dissolution of total phenols also increased, which is consistent with some previous reports [27], in line with the characteristics of phenolic substances being mainly dissolved in medium and high polarity solvents. Especially, MCLW contained hydroferulic acid, kaempferol 3-glucuronide, and (±)-3′,4′-methylenedioxy-5,7-dimethylepicatechin at much higher concentrations than MCLE and MCLH (Figure 3A–C, Table 1). Triterpenoid saponins (Momordicinin, Momordicine III, Momordicin I, Momordicoside F1, Momordicin II, Momordicoside K, Momordicoside F2 and Momoridin B) were mainly concentrated in MCLE.

### 3.3. Effects of MCLW on Body Composition and Blood Biochemical Parameters

**Figure 4:**
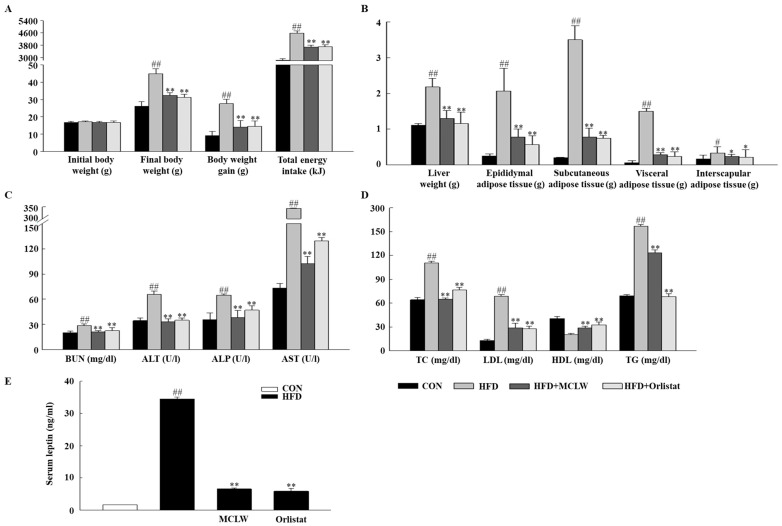
Effects of MCLW on body weight (**A**), liver and adipose tissue weights (**B**), biochemical parameters (**C**), lipid level (**D**), and serum leptin (**E**) in HFD-induced obese mice. Data are mean ± SEM. *n* = 8, Student’s unpaired *t*-test with Benjamini–Hochberg correction, ^#^ *p* < 0.05, ^##^ *p* < 0.01 vs. the normal diet (CON); * *p* < 0.05, ** *p* < 0.01 vs. the HFD. BUN, blood urea nitrogen; ALT, alanine aminotransferase; ALP, alkaline phosphatase; AST, aspartate aminotransferase; TC, total cholesterol; LDL, low-density lipoprotein cholesterol; HDL, high-density lipoprotein cholesterol; TG, triglycerides.

HFD feeding substantially increased adiposity and weight gain compared with the normal diet (Figure 4A). Increased liver weight, and white adipose tissue (visceral, subcutaneous, and epididymal) versus the control group confirmed the development of fat mass due to the HFD. MCLW or orlistat treatment substantially decreased the weight of the white adipose tissue (Figure 4B).

In addition, a blood biochemical analysis was conducted to determine the impact of MCLW on the biochemical parameters. In the biochemical analysis, the HFD group had significantly higher ALT, AST, ALP, BUN, TC, TG, and LDL than those of the CON group (Figure 4C,D). Compared to the HFD group, BUN, ALT, AST, and ALP were significantly reduced in MCLW or orlistat groups (Figure 4C). The MCLW or orlistat group had significantly reduced serum levels of TC, LDL, and TG compared to the HFD group (Figure 4D), whereas HDL levels in the MCLW or orlistat group increased in comparison with HFD group. Moreover, the leptin concentration in the MCLW or orlistat groups demonstrated a highly significant reduction in the HFD group (Figure 4E). These results are consistent with previous studies that plasma adipokine amounts are closely linked to lipid and control of energy intake by leptin [28]. The volume of serum was a limiting factor in these studies; future studies can determine whether MCLW improves other serum factor related to metabolic syndrome, such as serum adiponectin. The results suggest that serum leptin might be one of key players in reducing reduce the value of the fat tissue.

**Figure 5:**
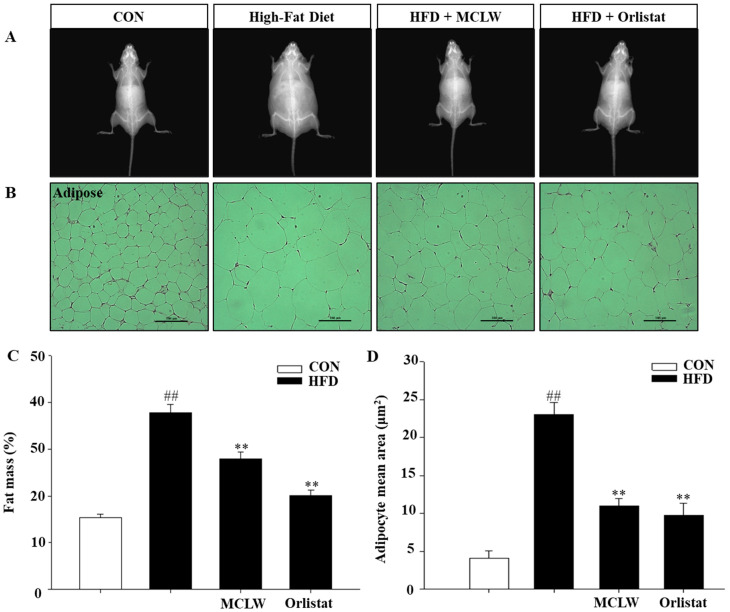
Changes in fat mass and adipocyte size in mice from normal and HFD diets groups, CON groups were fed normal diet, HFD groups fed high fat diet, HFD with MCLW 200 mg/kg, and HFD with Orlistat 60 mg/kg. (**A**) The radiography of body fat is displayed. (**B**) MCLW reduces lipid accumulation in the adipose tissue. (**C**) Fat mass values are reported relative to the body weight (*n* = 8 per group). (**D**) Adipocyte mean area (μm^2^) (*n* = 3 per group). Data are mean ± SEM. Student’s unpaired *t*-test with Benjamini–Hochberg correction, ^##^ *p* < 0.01 vs. the normal diet (CON); ** *p* < 0.01 vs. the HFD (*n* = 8).

The effects of MCLW on fat mass were calculated with DXA. The four-week MCLW supplement significantly reduced fat mass and adipocyte mean area in the obese mice that were induced by HFD (Figure 5; *p* < 0.01).

**Figure 6:**
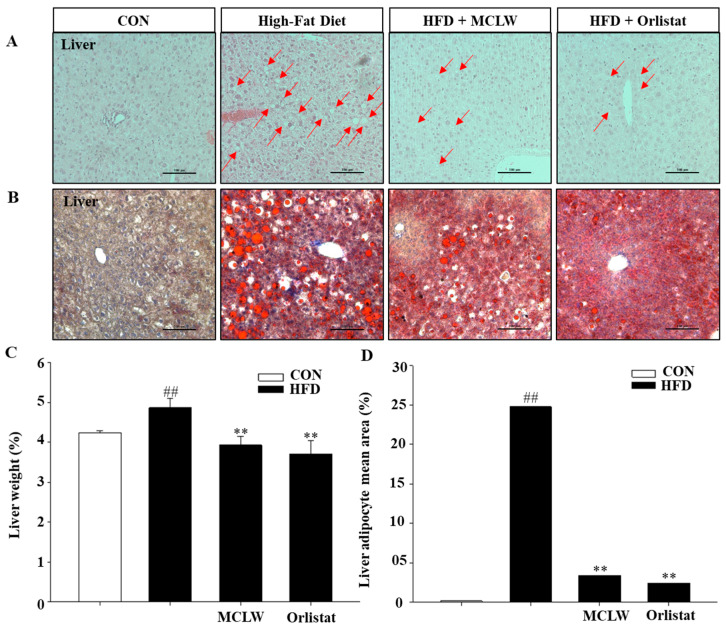
Histological analysis in liver on by H&E staining (**A**), Oil-Red O staining (**B**), and liver weight (**C**), (*n* = 8 per group). Liver adipocyte mean area (**D**) values are reported relative to the body weight (*n* = 3 per group). Data are mean ± SEM. Student’s unpaired *t*-test with Benjamini–Hochberg correction, ^##^ *p* < 0.01 vs. the normal diet (CON); ** *p* < 0.01 vs. the HFD (*n* = 8).

We also conducted a histological analysis of the liver by Oil-Red O staining and H&E staining to properly understand the reduced fat mass seen with the MCLW. Following the anti-obesity and hepatoprotective effects predicted, the results of the study showed that lipid accumulation in liver had increased significantly in HFD group (Figure 6). On the contrary, MCLW or orlistat supplementation significantly reduced the adipocyte region of liver in the MCLW or orlistat group (Figure 6).

### 3.4. Effects of MCLW on Fat Metabolic Pathways

**Figure 7:**
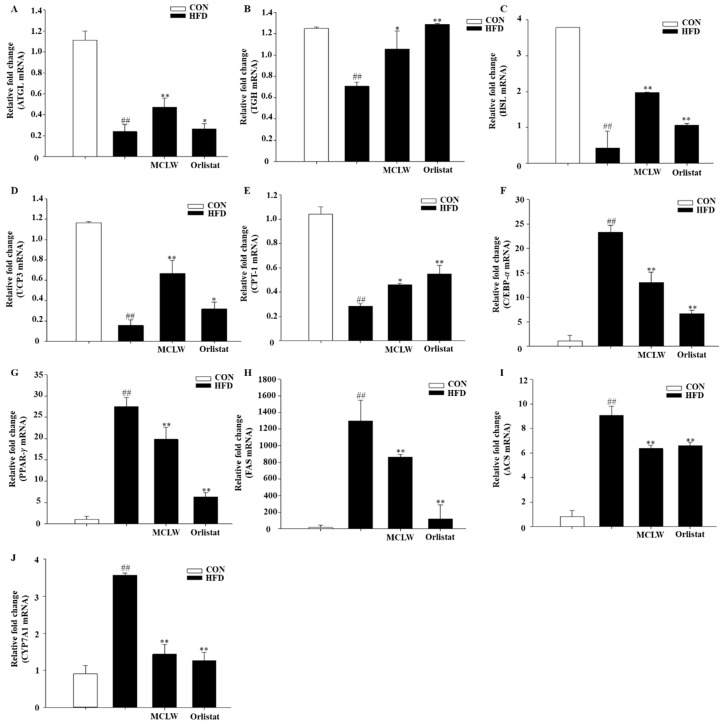
mRNA expression in mouse liver as measured in real time PCR. The charts showed expression levels with ATGL (**A**), TGH (**B**), HSL (**C**), UCP3 (**D**), CPT-1 (**E**), C/EBP-α (**F**), PPAR-γ (**G**), FAS (**H**), ACS (**I**), CYP7A1 (**J**). All the data represented the relative fold change of mRNA expression of the genes of interest. The fold-change for each gene is relative to CON mRNA levels. Data are mean ± SEM. *n* = 8, Student’s unpaired *t*-test with Benjamini–Hochberg correction, ^##^ *p* < 0.01 vs. the CON. * *p* < 0.05, ** *p* < 0.01 vs. the HFD (*n* = 3).

**Figure 8:**
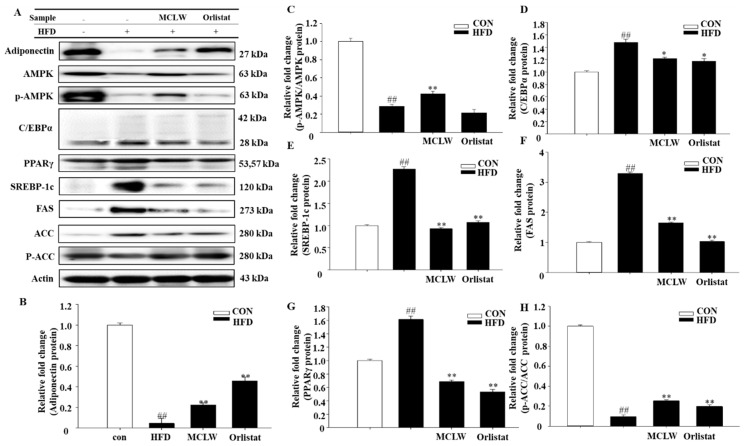
Western blot (**A**). Adiponectin (**B**), p-AMPK/AMPK (**C**), C/EBPα (**D**), PPAR-γ (**E**), SREBP-1c (**F**), FAS (**G**), and p-ACC/ACC (**H**) protein expression levels, normalized relative protein expression data to actin expression levels. Data are mean ± SEM. *n* = 3, Student’s unpaired *t*-test with Benjamini–Hochberg correction, ^##^ *p* < 0.01 vs. the CON. * *p* < 0.05, ** *p* < 0.01 vs. the HFD.

We measured the mRNA and protein levels of AMPK, adipose triglyceride lipase (ATGL), triacylglycerol hydrolase (TGH), hormone-sensitive lipase (HSL), carnitine palmitoyl transferase I (CPT-I), and porcine uncoupling protein 3 (UCP3). MCLW increased the fatty acid metabolic mRNA and protein levels, as indicated by ATGL, TGH, HSL, UCP3, and CPT-1 mRNA levels (Figure 7A–E), and adiponectin and AMPK protein levels in HFD+MCLW group (Figure 8A–C).

Moreover, we analyzed the impact with MCLW on mRNA and protein concentrations with lipogenic enzymes and their regulators, including CCAAT/enhancer binding protein α (C/EBP-α), SREBP-1c, peroxisome proliferator-activated receptor γ (PPAR-γ), cholesterol 7α-hydroxylase (CYP7A1), acyl-CoA synthetase (ACS), ACC, and FAS. MCLW treatment led to reduction of mRNA levels for C/EBPα, PPARγ, FAS, ACS, and CYP7A1 (Figure 7F–J), as well as protein levels involving C/EBPα, SREBP-1c, PPARγ, FAS (Figure 8). The MCLW group had higher levels of expression of p-AMPK and p-ACC protein, indicating that MCLW regulating the expression of key molecules in the AMPK pathway (Figure 8). 

## 4. Discussion

Obesity causes systemic oxidative stress via a variety of biochemical pathways, including reduced antioxidant defenses [29]. Furthermore, oxidative stress can stimulate the proliferation, differentiation, maturation of fat cells, and the size of fat cells can increase fat accumulation [30]. Many phytochemical antioxidants, including vitamin C, carotenoids, vitamin E have the capacity to act as antioxidants, minimize oxidative stress in the body [31]. In our study, the fruit, root, stem, and leaf of *M. charantia* were extracted with distilled water, 70% ethanol, and 95% hexane. In total, 12 extracts were obtained. Our findings showed that antioxidant activities with MCLW and MCLE were significantly higher than that of MCLH. Therefore, they were selected for further evaluation. MCLW exhibited the highest antiadipogenic potential on 3T3-L1 preadipogenic cells. Therefore, we conducted in vivo experiments to explore the anti-obesity mechanism of MCLW.

The results of this experiment clearly indicate that the MCLW inhibits fat deposition in HFD-fed mice by regulating lipid metabolism. In this experiment, HFD caused increased energy intake and fat accumulation in adipose and liver tissues, thus resulting in increased body mass. MCLW effectively inhibited the increase in energy intake, body weight, and white adipose tissue in mice. Compared to the HFD group, MCLW *p.o* for four weeks significantly reduced serum TG, TC, and LDL contents, and increased HDL content, thereby reducing the liver weight. This suggests that MCLW can prevent elevated blood lipid levels induced by HFD. Lipid accumulation was found to be reduced in the liver tissue, and the cross-section area of adipocytes in adipose tissue was significantly reduced. Therefore, the fat weight and volume were reduced significantly in mice fed with MCLW compared to HFD-fed mice.

Consistent with the antioxidant results, triterpenoid saponins like momordicine III content in MCLW and MCLE was higher than MCLH (Figure 3A–C, Table 1). Many types of saponins have been isolated from *M. charantia*, including Momordicinin, Momordicine III, Momordicin I, Momordicoside F1, Momordicin II, Momordicoside K, Momordicoside F2, and Momoridin B [32]. These ingredients have been found to reduce visceral fat weight and glucose levels, promote oxidation in liver and adipose tissue, and significantly lower blood TG levels [33,34,35]. Meanwhile, in MCLE, triterpenoid saponins were much higher than MCLW (Figure 3A–C, Table 1), conflicts with the results of the 3T3-L1 adipocytes experiment. Therefore, we assumed that there were other main ingredients that suppressed obesity in MCLW. According to the results of composition analysis, we found that the types and contents of polyphenols in MCLW were significantly higher than those in MCLE. The structure of (±)-3′,4′-Methylenedioxy-5,7-dimethylepicatechin is very similar to the structure of catechin, so (±)-3′,4′-Methylenedioxy-5,7-dimethylepicatechin may function like a catechin analog, leading to the activation of AMPK, which suppresses the expression of genes encoding enzymes and transcription factors involved in adipogenesis and lipogenesis, and stimulates those involved in lipolysis [36,37]. This is consistent with our findings, as *M. charantia* extracts showed significant fatty acid metabolism regulating capacity. Kaempferol is a flavonol present in many traditional medicines and edible plants [38]. It has been suggested that kaempferol can reduce body weight and adipose tissue [39]. In our experiment, MCLW had a certain hypoglycemic effect. HPLC chromatogram showed that hydroferulic acid was present in MCLW (5.57 ng/g), MCLE (2.59 ng/g), and MCLH (1.96 ng/g) samples (Figure S2A–C). The results revealed that hydroferulic acid was much higher in MCLW than MCLE and MCLH (Figure S2A–C, Table 1). Hydroferulic acid is a derivative of chlorogenic acid, and the structure of the two is similar [40]. By protecting animals from chemical and lipopolysaccharide attacks, chlorogenic acid has hepatoprotective effects [41,42]. Chlorogenic acid′s anti-obesity effects can be attributed to impaired nutrient metabolism, including that for glucose and fatty acids [43,44]. These results suggest that the anti-obesity activities of MCLW possibly related to hydroferulic acid, kaempferol 3-glucuronide, and (±)-3′,4′-methylenedioxy-5,7-dimethylepicatechin. Nevertheless, since we administered crude extract, MCLW, our research did not determine the key component for the anti-obesity effect among the listed compositions. Further study is required to determine the main functional compound for the anti-obesity effect in obese mice induced with HFD.

Fat cells are central to the accumulation of surplus energy in the form of TG. Preadipocytes in turn are transformed into mature adipocytes by elevated energy intake, leptin, and glucocorticoids [45,46]. Therefore, targeting fat cells is the key to treating obesity. The differentiation of preadipocytes into adipocytes is typically a complex process involving changes in the morphology of cells, gene expression, hormone sensitivity, and lipogenic transcription factors. The accumulation process is exceptionally crucial [47]. During the differentiation of preadipocytes into mature adipocytes, they are regulated by numerous transcription factors. PPAR-γ and C/EBP-α act together to initiate the expression of fat-specific genes, thereby promoting the production and accumulation of long-chain fatty acids [48,49]. PPAR-γ upregulates CYP7A1 in hepatocytes and promotes cholesterol formation [50]. The results revealed that MCLW administration inhibited the expression of C/EBP-α, PPAR-γ, and CYP7A1, decreased the formation of fatty acids and cholesterol, and inhibited the differentiation of preadipocytes into adipocytes compared with HFD-fed mice.

Throughout the late stage of differentiation, fat cells secrete adiponectin, leptin, and other adipose tissue-specific products to accelerate fat accumulation [51]. In this study, MCLW was introduced to effect two important metabolism adipokines: adiponectin and leptin. Adiponectin facilitates many physiological functions, like oxidation of fatty acids, and regulation of glucose [52]. This part about glucose metabolism was also confirmed in this experiment; MCLW could effectively improve the glucose regulation of HFD-fed mice (Figure S3). Therefore, increased adiponectin concentration increases the lipid catabolism, demonstrating that MCLW administration results in hypolipidemic activity, at least in part by regulating these adipokines.

After differentiation, the fat accumulation depends on TG breakdown and fatty acid synthesis to regulate lipid metabolism [53]. This study found that MCLW can significantly upregulate AMPK levels in adipose tissue and inhibit fatty acid synthesis. SREBP-1c is a downstream molecule regulated by AMPK. It mainly activates a series of genes related to fat synthesis (ACC and FAS), thereby regulating fatty acid metabolism [54,55]. FAS activity directly controls the strength of fat synthesis, and the increased gene expression level can significantly increase the deposition of TG [56], which is proven by the results of the present investigation. ACS is one of the main target genes of FAS. We identified the phosphorylation level of ACC, another lipid synthesis-related enzyme regulated by AMPK along with FAS [57]. Previous studies have confirmed that increased ACC activity can promote the synthesis and deposition of cellular TG [58], which is in accordance with the results of this test. CPT1 is the main enzyme for mitochondrial fatty acid β-oxidation and can be inhibited by ACC. When AMPK is phosphorylated and activated, ACC, an enzyme that turns acetyl-CoA into malonyl-CoA, is inactivated by phosphorylation, thereby promoting CPT1-regulated reducing lipid deposition and fatty acid beta-oxidation in the peripheral tissues [59]. Compared to HFD groups, as in Figure 8, p-AMPK and p-ACC are significantly higher in MCLW group. This indicates that MCLW may inhibit lipid metabolism by regulating the expression of key molecules in the AMPK pathway. In this analysis, levels of UCP3 and CPT1 in adipose tissue were lower, and levels of SREBP1c and FAS protein were higher in the HFD group, than those in the CON group. The MCLW group had higher levels of UCP3 and CPT1 than those in the HFD group. In the MCLW or orlistat group, the level of expression of SREBP1c and FAS protein was lower than that of the HFD group. These data suggest that MCLW regulates fatty acid oxidation through the activation of AMPK and the deactivation of ACC, thereby impeding TG deposition in cells.

The presence of TG is the result of a dynamic equilibrium with synthesis and decomposition, which undergoes decomposition during synthesis. ATGL, TGH, and HSL are also the major capable of catalyzing the breakdown of TG fat in liver tissue [60]. ATGL catalyzes, in particular, the initial step of TG hydrolysis for the processing of DG [61]. HSL catalyzes DG hydrolysis in the second phase of the lipolysis cycle, and is the most crucial enzyme in fat breakdown [62]. In this experiment, the contents of in MCLW group, ATGL and HSL have been considerably higher than those in the HFD group, indicating that MCLW could substantially increase ATGL and HSL mRNA expression. TGH is present in the cytoplasm, lipid droplets, and cell membranes, and is an important lipase that catalyzes the hydrolysis of TG, thus contributing to non-HSL-mediated TG lipolysis in the adipocytes [63]. Our results indicate that MCLW upregulates liver TGH mRNA expression and promotes TG decomposition. Uncoupling protein 3 (UCP3) promotes fatty acid oxidation and affects the body weight, body fat, and resting metabolic rate [64]. Literature reveals that AMPK can control production of UCP3 in muscle tissue [65]. Our results demonstrate that MCLW upregulates UCP3 mRNA expression and enhances energy metabolism regulation in liver tissue. This suggests that MCLW may indirectly promote TG decomposition.

In this study, it was found that MCLW had a significant improvement effect on obesity and obesity-induced lipid metabolism, but the effect of MCLW was slightly weaker than that of orlistat. It is worth noting that MCLW was superior to orlistat in AST and ALP. This suggests that MCLW is more effective than orlistat in improving liver and kidney injury.

## 5. Conclusions

**Figure 9:**
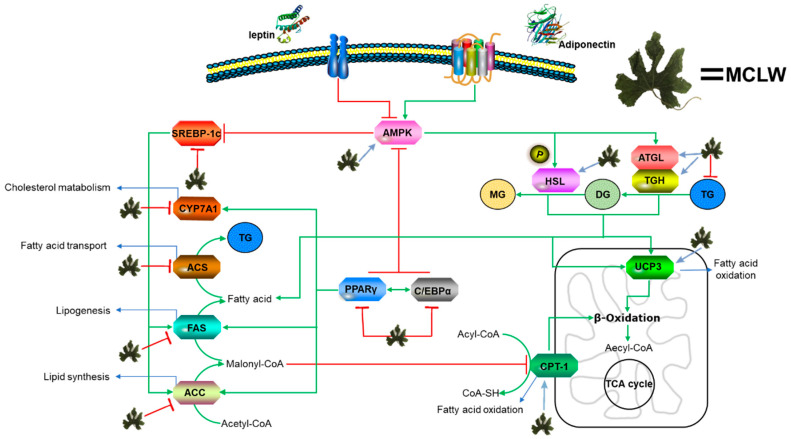
Outline of a working hypothesis for fat metabolic pathways in HFD-fed mice. MCLW increases lipolysis by regulating the expression of AMPK and associated downstream signaling molecules.

In this study, we assessed the antioxidant activity of 12 extracts obtained from different parts of *M. charantia* using different solvents. MCLW was found to reduce body weight, and energy intake of HFD-fed mice, and inhibited 3T3-L1 preadipocyte differentiation. Furthermore, MCLW inhibited the gene expression related to synthesis of TG in HFD-fed mice, promoted decomposition of TG, and reduced the fat content and deposition in mouse adipose tissue and liver. This relates to the expressional regulation of key molecules in multiple signaling pathways for lipid metabolism (Figure 9). From the results, MCLW might be considered as a dietary supplement for treating obesity. 

## Figures and Tables

**Table 1:** List of biomarkers to discriminate MCLW, MCLE, and MCLH.

Peak No.	Retention Time (min)	Identification (MCLW)	Exact Mass (*m*/*z*)	Fragment Ions (*m*/*z*)
1	0.68	Hydroferulic acid	195.08	165, 133
2	0.91	*N*-(1-Deoxy-1-fructosyl)leucine	292.14	274
3	1.06	Chlorogenic acid fragments	353.08	323, 191, 129
4	1.37	Abscisic acid fragments	263.13	188, 144
5	1.43	Hyperforin fragments	535.37	257, 243
6	1.70	Rosmaricine	344.09	150
7	1.85	Biotin fragments	243.08	200, 156
8	2.18	*N*-[2-Hydroxy-2-(4-hydroxyphenyl)ethyl]cinnamide	282.12	150, 133
9	2.73	Kaempferol 7-arabinoside	417.16	357, 215, 135
10	2.91	alpha,alpha-Trehalose	341.14	135, 119
11	3.19	Kaempferol 3-glucuronide	461.14	399, 285
12	3.36	Orientin 7,3′-dimethyl ether	475.16	445, 299
13	3.53	Momordicinin	437.26	421, 409, 205
14	3.67	5-(6-Hydroxy-3,7-dimethyl-2,7-octadienyloxy)-7-methoxycoumarin	343.26	327, 301
15	3.76	Phytolaccasaponin G	663.47	645, 601, 501
16	4.06	Phytolaccasaponin G	663.47	645, 631, 485
17	4.21	(±)-3′,4′-Methylenedioxy-5,7-dimethylepicatechin	329.28	229
18	4.34	Myrianthic acid	503.41	487, 485
19	4.73	Momordicine III	647.47	485, 99
20	4.93	Goyaglycoside b	647.47	485
21	4.96	5beta,19-Epoxy-19-methoxycucurbita-6,23(E)-diene-3beta,25-diol	485.40	469, 455
22	5.20	Momordicin I	471.42	99
23	5.48	5beta,19-Epoxy-19-methoxycucurbita-6,23(E)-diene-3beta,25-diol	485.40	455, 325
24	5.54	Madasiatic acid	487.42	471
25	5.69	Vernolic acid	295.27	277, 195, 99
**Peak** **No.**	**Retention Time** **(min)**	**Identification (MCLE)**	**Exact Mass** **(*m*/*z*)**	**Fragment Ions** **(*m*/*z*)**
1	0.83	Isorhamnetin 7-rhamnoside	461.13	357, 285, 283
2	0.98	Diosmetin 7-glucuroside	475.15	445, 299, 269
3	1.48	6-Hydroxyluteolin 7,3′,4′-trimethyl ether	343.25	327, 283, 243
4	1.58	Apigenin 6-C-arabinosyl-8-C-glucoside	695.49	663, 519
5	1.69	Luteolin	285.08	269, 135
6	1.87	ent-Epiafzelechin(2a->7,4a->8)epiafzelechin 3-(4-hydroxybenzoic acid)	663.46	645
7	2.13	3,3′,5-Trihydroxy-4′-methoxy-6,7-methylenedioxyflavone 3-glucuronide	519.40	501, 343, 327
8	2.33	Apigenin	269.07	227, 151, 117
9	2.41	5-Hydroxy-7,3′,4′-trimethoxy-8-methylisoflavone 5-neohesperidoside	649.48	631, 487
10	2.46	Diosmetin	299.06	284, 256
11	2.81	5,7-Dihydroxy-8,4′-dimethoxyisoflavone	329.27	229, 139
12	3.04	Phytolaccoside E	825.57	617, 487
13	3.36	5beta,19-Epoxy-19-methoxycucurbita-6,23(E)-diene-3beta,25-diol	485.39	455
14	3.80	Goyaglycoside b	647.46	631, 485, 455
15	3.89	Momordicoside F1	631.47	469
16	4.05	Goyaglycoside c	661.45	645, 631, 499
17	4.14	Momordicin II	633.45	615, 471, 455
18	4.34	Momordicine III	647.46	617, 485
19	4.45	Momordicoside K	647.46	617
20	4.61	Dihydroxystearic acid	315.29	297
21	4.76	Momordicoside F2	617.45	527, 455
22	4.90	Momoridin B	631.47	469, 455
23	5.33	Nonadecanoic acid	297.28	279
24	6.21	1,17-Heptadecanediol	271.26	225
25	6.82	Retinoic acid	299.30	253, 99
**Peak** **No.**	**Retention Time** **(min)**	**Identification (MCLH)**	**Exact Mass** **(*m*/*z*)**	**Fragment Ions** **(*m*/*z*)**
1	1.17	Biotin	243.15	225, 199
2	1.48	Biotin	243.15	225, 197
3	1.97	Cetyl alcohol	241.13	197, 127, 125, 99
4	2.81	5,7-Dihydroxy-8,4′-dimethoxyisoflavone	329.27	229
5	3.36	3,3′,4′,5,6,8-Hexamethoxyflavone	401.23	375, 329, 313, 209
6	3.96	5,7-Dihydroxy-3′,4′-dimethoxy-5′-prenylflavanone	383.22	353, 311
7	4.04	2′,3,4′,5-Tetrahydroxy-4-prenylstilbene	311.26	293, 265
8	4.18	Cycloheterophyllin	501.38	461, 311
9	4.46	Medicarpin 3-O-(6′-malonylglucoside)	517.38	499, 487, 471, 311
10	4.66	Limocitrin 3-rhamnoside	491.40	475, 371, 313
11	4.98	Phytal	293.24	277, 263
12	5.36	Vernolic acid	295.26	279
13	5.86	Madasiatic acid	487.40	471, 425
14	5.99	5beta,19-Epoxy-19-methoxycucurbita-6,23(E)-diene-3beta,25-diol	485.38	455, 427, 423
15	6.23	5beta,19-Epoxy-19-methoxycucurbita-6,23(E)-diene-3beta,26-diol	485.38	455
16	6.54	Myrianthic acid	503.40	487

## Data Availability

The data presented in this study are available on request from the corresponding author.

## Supplementary Materials

The following are available online at https://www.mdpi.com/article/10.3390/ijerph18115584/s1, Figure S1: MCLD and MCLE had no cytotoxic effect on 3T3-L1, Figure S2: HPLC Chromatogram of Hydroferulic Acid in MCLD, MCLE and MCLH, Figure S3: MCLD treatment improved metabolic parameters of diabetes and obesity in high-fat diet fed mice, Table S1: Primers used in the reverse transcriptase-polymerase chain reaction analysis.

## References

[1] Mokdad A.H. (2018). Burden of Obesity in the Eastern Mediterranean Region: Findings from the Global Burden of Disease 2015 Study. Int. J. Environ. Res. Public Health.

[2] Bhadoria A.S., Sahoo K., Sahoo B., Choudhury A.K., Sofi N.Y., Kumar C.A. (2015). Childhood Obesity: Causes and Consequences. J. Fam. Med. Prim. Care.

[3] Pascual-Serrano A., Arola-Arnal A., Suárez-García S., Bravo F.I., Suárez M., Arola L., Bladé C. (2017). Grape Seed Proanthocya-nidin Supplementation Reduces Adipocyte Size and Increases Adipocyte Number in Obese Rats. Int. J. Obes..

[4] Krentz A.J., Fujioka K., Hompesch M. (2016). Evolution of Pharmacological Obesity Treatments: Focus on Adverse Side-effect Profiles. Diabetes Obes. Metab..

[5] Annamalai S., Mohanam L., Alwin D., Prabhu V. (2016). Effect of Combination Therapy of Melatonin and Orlistat on High Fat Diet Induced Changes in Lipid Profiles and Liver Function Parameters in Serum of Rats. Obes. Med..

[6] Wang L.-C., Pan T.-M., Tsai T.-Y. (2018). Lactic Acid Bacteria-fermented Product of Green Tea and Houttuynia Cordata Leaves Exerts anti-adipogenic and Anti-obesity Effects. J. Food Drug Anal..

[7] Bai J., Zhu Y., Dong Y. (2016). Response of Gut Microbiota and Inflammatory Status to Bitter Melon (*Momordica charantia* L.) in High Fat Diet Induced Obese Rats. J. Ethnopharmacol..

[8] Daniel P., Supe U., Roymon M.G. (2014). A Review on Phytochemical Analysis of *Momordica charantia*. Int. J. Adv. Pharm. Biol. Chem..

[9] Jia S., Shen M., Zhang F., Xie J. (2017). Recent Advances in *Momordica charantia*: Functional Components and Biological Activities. Int. J. Mol. Sci..

[10] Annapoorani C.A., Manimegalai K. (2013). Screening of Medicinal Plant *Momordica charantia* Leaf for Secondary Metabolites. Int. J. Pharm. Res. Dev..

[11] Shivanagoudra S.R., Perera W.H., Perez J.L., Athrey G., Sun Y., Jayaprakasha G., Patil B.S. (2019). Cucurbitane-type Compounds from *Momordica charantia*: Isolation, In Vitro Antidiabetic, Anti-inflammatory Activities and in Silico Modeling Approaches. Bioorganic Chem..

[12] Farah N., Bukhari S.A., Ali M., Naqvi S.A.R., Mahmood S. (2018). Phenolic Acid Profiling and Antiglycation Studies of Leaf and Fruit Extracts of Tyrosine Primed *Momordica charantia* Seeds for Possible Treatment of Diabetes Mellitus. Pak. J. Pharm. Sci..

[13] Krawinkel M.B., Keding G.B. (2006). Bitter Gourd (*Momordica charantia*): A Dietary Approach to Hyperglycemia. Nutr. Rev..

[14] Yoon N.A., Park J., Lee J., Jeong J.Y., Kim H.-K., Lee H.S., Hwang I.G., Roh G.S., Kim H.J., Cho G.J. (2017). Anti-diabetic Effects of Ethanol Extract from Bitter Melon in Mice Fed a High-fat Diet. Dev. Reprod..

[15] Grover J.K., Yadav S.P. (2004). Pharmacological Actions and Potential Uses of *Momordica charantia*: A Review. J. Ethnopharmacol..

[16] Blois M.S. (1958). Antioxidant Determinations by the Use of a Stable Free Radical. Nat. Cell Biol..

[17] Oyaizu M. (1986). Studies on Products of Browning Reaction Antioxidative Activities of Products of Browning Reaction Prepared from Glucosamine. Jpn. J. Nutr. Diet..

[18] Rudich A., Tirosh A., Bashan N. (1998). Prolonged Oxidative Stress Impairs Insulin-induced GLUT4 Translocation in 3T3-L1 Adipo-cytes. Diabetes.

[19] Fan M., Choi Y.-J., Tang Y., Bae S.M., Yang H.P., Kim E.-K. (2019). Efficacy and Mechanism of Polymerized Anthocyanin from Grape-Skin Extract on High-Fat-Diet-Induced Nonalcoholic Fatty Liver Disease. Nutrients.

[20] Choi Y.-J., Fan M., Tang Y., Yang H.P., Hwang J.-Y., Kim E.-K. (2019). In Vivo Effects of Polymerized Anthocyanin from Grape Skin on Benign Prostatic Hyperplasia. Nutrients.

[21] Matsuda M., Shimomura I. (2013). Increased Oxidative Stress in Obesity: Implications for Metabolic Syndrome, Diabetes, Hypertension, Dyslipidemia, Atherosclerosis, and Cancer. Obes. Res. Clin. Pract..

[22] Fernández-Sánchez A., Madrigal-Santillán E., Bautista M., Esquivel-Soto J., Morales-González Á., Esquivel-Chirino C., Durante-Montiel I., Sánchez-Rivera G., Valadez-Vega C., Morales-González J.A. (2011). Inflammation, Oxidative Stress, and Obesity. Int. J. Mol. Sci..

[23] Verdile G., Keane K.N., Cruzat V.F., Medic S., Sabale M., Rowles J., Wijesekara N., Martins R.N., Fraser P.E., Newsholme P. (2015). Inflammation and Oxidative Stress: The Molecular Connectivity between Insulin Resistance, Obesity, and Alzheimer’s Disease. Mediat. Inflamm..

[24] Nijhawan P., Arora S., Behl T. (2019). Intricate role of oxidative stress in the progression of obesity. Obes. Med..

[25] Amin M.N., Siddiqui S.A., Uddin G., Ibrahim M., Uddin S.M.N., Adnan T., Rahaman Z., Kar A., Islam M.S. (2020). Increased Oxidative Stress, Altered Trace Elements, and Macro-Minerals Are Associated with Female Obesity. Biol. Trace Elem. Res..

[26] Di Domenico M., Pinto F., Quagliuolo L., Contaldo M., Settembre G., Romano A., Nicoletti G.F. (2019). The Role of Oxidative Stress and Hormones in Obesity. Front. Endocrinol..

[27] Shafazila T.S., Lee P.M., Hung L.K. Radical Scavenging Activities of Extract and Solvent-solvent Partition Fractions from Dendrobium Sonia “Red Bom” Flower. Proceedings of the 2010 International Conference on Science and Social Research, CSSR 2010.

[28] Sun Y., Yang Y., Qin Z., Cai J., Guo X., Tang Y., Wan J., Su D.-F., Liu X. (2016). The Acute-Phase Protein Orosomucoid Regulates Food Intake and Energy Homeostasis via Leptin Receptor Signaling Pathway. Diabetes.

[29] Saltiel A.R., Olefsky J.M. (2017). Inflammatory Mechanisms Linking Obesity and Metabolic Disease. J. Clin. Investig..

[30] Chimin P., Andrade M.L., Belchior T., Paschoal V.A., Magdalon J., Yamashita A.S., Castro É., Castoldi A., Chaves-Filho A.B., Yoshinaga M.Y. (2017). Adipocyte mTORC1 Deficiency Promotes Adipose Tissue Inflammation and NLRP3 Inflammasome Activation via Oxidative Stress and de Novo Ceramide Synthesis. J. Lipid Res..

[31] Septembre-Malaterre A., Stanislas G., Douraguia E., Gonthier M.-P. (2016). Evaluation of Nutritional and Antioxidant Properties of the Tropical Fruits Banana, Litchi, Mango, Papaya, Passion Fruit and Pineapple Cultivated in Réunion French Island. Food Chem..

[32] Fan M., Kim E.-K., Choi Y.-J., Tang Y., Moon S.-H. (2019). The Role of *Momordica charantia* in Resisting Obesity. Int. J. Environ. Res. Public Health.

[33] Kalaivani A., Uddandrao V.V.S., Brahmanaidu P., Saravanan G., Nivedha P.R., Tamilmani P., Swapna K., Vadivukkarasi S. (2018). Anti Obese Potential of Cucurbita Maxima Seeds Oil: Effect on Lipid Profile and Histoarchitecture in High Fat Diet Induced Obese Rats. Nat. Prod. Res..

[34] Brusaferro A., Cozzali R., Orabona C., Biscarini A., Farinelli E., Cavalli E., Grohmann U., Principi N., Esposito S. (2018). Is It Time to Use Probiotics to Prevent or Treat Obesity?. Nutrients.

[35] Ghaedi E., Varkaneh H.K., Rahmani J., Mousavi S.M., Mohammadi H., Fatahi S., Pantovic A., Mofrad M.D., Zhang Y. (2019). Possible Anti-obesity Effects of Phytosterols and Phytostanols Supplementation in Humans: A Systematic Review and Dose–Response Meta-analysis of Randomized Controlled Trials. Phytother. Res..

[36] Hossain M.A., Weli A.M., Ahmed S.H.I. (2019). Comparison of Total Phenols, Flavonoids and Antioxidant Activity of Various Crude Extracts of Hyoscyamus Gallagheri Traditionally Used for the Treatment of Epilepsy. Clin. Phytoscience.

[37] Jiang Y., Ding S., Li F., Zhang C., Sun-Waterhouse D., Chen Y., Li D. (2019). Effects of (+)-catechin on the Differentiation and Lipid Metabolism of 3T3-L1 Adipocytes. J. Funct. Foods.

[38] M Calderon-Montano J., Burgos-Morón E., Pérez-Guerrero C., López-Lázaro M. (2011). A Review on the Dietary Flavonoid Kaempferol. Mini Rev. Med. Chem..

[39] Zhou W., Shan J., Meng M. (2018). A Two-step Ultra-high-performance Liquid Chromatography-Quadrupole/Time of Flight Mass Spectrometry with Mass Defect Filtering Method for Rapid Identification of Analogues from Known Components of Different Chemical Structure Types in Fructus Gardeniae-Fructus Forsythiae Herb Pair Extract and in Rat’s Blood. J. Chromatogr. A.

[40] Baeza G., Bachmair E.M., Wood S., Mateos R., Bravo L., De Roos B. (2017). The Colonic Metabolites Dihydrocaffeic Acid and Di-hydroferulic Acid are more Effective Inhibitors of In Vitro Platelet Activation than their Phenolic Precursors. Food Funct..

[41] Naveed M., Hejazi V., Abbas M., Kamboh A.A., Khan G.J., Shumzaid M., Ahmad F., Babazadeh D., FangFang X., Modarresi-Ghazani F. (2018). Chlorogenic acid (CGA): A Pharmacological Review and Call for Further Research. Biomed. Pharmacother..

[42] Wang Q., Lu K., Li F., Lei L., Zhao J., Wu S., Yin R., Ming J. (2019). Polyphenols from Morchella Angusticepes Peck Attenuate D-galactosamine/Lipopolysaccharide-induced Acute Hepatic Failture in Mice. J. Funct. Foods.

[43] Wang Z., Lam K.L., Hu J., Ge S., Zhou A., Zheng B., Lin S. (2019). Chlorogenic Acid Alleviates Obesity and Modulates Gut Microbiota in High-fat-fed Mice. Food Sci. Nutr..

[44] Chen L., Teng H., Cao H. (2019). Chlorogenic Acid and Caffeic Acid from Sonchus Oleraceus Linn Synergistically Attenuate Insulin Resistance and Modulate Glucose Uptake in HepG2 Cells. Food Chem. Toxicol..

[45] Ghaben A.L., Scherer P.E. (2019). Adipogenesis and Metabolic Health. Nat. Rev. Mol. Cell Biol..

[46] Abulizi A., Camporez J.P., Jurczak M.J., Høyer K.F., Zhang D., Cline G.W., Samuel V.T., Shulman G.I., Vatner D.F. (2019). Ad-ipose Glucocorticoid Action Influences Whole-body Metabolism via Modulation of Hepatic Insulin Action. FASEB J..

[47] Moreno-Navarrete J.M., Fernández-Real J.M., Metzler J.B. (2017). Adipocyte Differentiation. Adipose Tissue Biology.

[48] Choi S.K., Park S., Jang S., Cho H.H., Lee S., You S., Kim S.-H., Moon H.-S. (2016). Cascade Regulation of PPARγ2 and C/EBPα Signaling Pathways by Celastrol Impairs Adipocyte Differentiation and Stimulates Lipolysis in 3T3-L1 Adipocytes. Metabolism.

[49] Kim N.H., Kim H.J., Im J.Y., Kwak W.R., Kim Y.H., Kim D.K., Lim S., Lee Y. (2019). Pulsatilla Koreana Nakai Downregulates C/EBPs/PPARγ and Suppresses Fatty Acid Synthase via Activation of AMPKα in 3T3-L1 Cells. Indian J. Pharm. Sci..

[50] Zhang J.-M., Wang X.-H., Hao L.-H., Wang H., Zhang X.-Y., Muhammad I., Qi Y., Li G.-L., Sun X.-Q. (2017). Nrf2 is Crucial for the Down-regulation of Cyp7a1 Induced by Arachidonic Acid in Hepg2 Cells. Environ. Toxicol. Pharmacol..

[51] Stern J.H., Rutkowski J.M., Scherer P.E. (2016). Adiponectin, Leptin, and Fatty Acids in the Maintenance of Metabolic Homeostasis through Adipose Tissue Crosstalk. Cell Metab..

[52] Ghadge A.A., Khaire A.A., Kuvalekar A.A. (2018). Adiponectin: A Potential Therapeutic Target for Metabolic Syndrome. Cytokine Growth Factor Rev..

[53] Goo Y.H., Son S.H., Paul A. (2017). Lipid Droplet-associated Hydrolase Promotes Lipid Droplet Fusion and Enhances ATGL Degradation and Triglyceride Accumulation. Sci. Rep..

[54] Chen K., Chen X., Xue H., Zhang P., Fang W., Chen X., Ling W. (2019). Coenzyme Q10 Attenuates High-fat Diet-induced Non-alcoholic Fatty Liver Disease through Activation of the AMPK Pathway. Food Funct..

[55] Lin K.-T., Hsu S.-W., Lai F.-Y., Chang T.-C., Shi L.-S., Lee S.-Y. (2016). Rhodiola Crenulata Extract Regulates Hepatic Glycogen and Lipid Metabolism via Activation of the AMPK Pathway. BMC Complement. Altern. Med..

[56] Angeles T.S., Hudkins R.L. (2016). Recent Advances in Targeting the Fatty Acid Biosynthetic Pathway Using Fatty Acid Synthase In-hibitors. Expert Opin. Drug Dis..

[57] Harriman G., Greenwood J., Bhat S., Huang X., Wang R., Paul D., Tong L., Saha A.K., Westlin W.F., Kapeller R. (2016). Acetyl-CoA Carboxylase Inhibition by ND-630 Reduces Hepatic Steatosis, Improves Insulin Sensitivity, and Modulates Dyslipidemia in Rats. Proc. Natl. Acad. Sci. USA.

[58] Saponaro C., Gaggini M., Carli F., Gastaldelli A. (2015). The Subtle Balance between Lipolysis and Lipogenesis: A Critical Point in Metabolic Homeostasis. Nutrients.

[59] Galic S., Loh K., Murray-Segal L., Steinberg G.R., Andrews Z.B., Kemp B.E. (2018). AMPK Signaling to Acetyl-CoA Carboxylase is Required for Fasting-and Cold-induced Appetite but not Thermogenesis. Elife.

[60] Morak M., Schmidinger H., Riesenhuber G., Rechberger G.N., Kollroser M., Haemmerle G., Zechner R., Kronenberg F., Hermetter A. (2012). Adipose Triglyceride Lipase (ATGL) and Hormone-Sensitive Lipase (HSL) Deficiencies Affect Expression of Lipolytic Activities in Mouse Adipose Tissues. Mol. Cell. Proteom..

[61] Kim S.J., Tang T., Abbott M., Viscarra J.A., Wang Y., Sul H.S. (2016). AMPK Phosphorylates Desnutrin/ATGL and Hor-mone-sensitive Lipase to Regulate Lipolysis and Fatty Acid Oxidation within Adipose Tissue. Mol. Cell Biol..

[62] Schulze R.J., Sathyanarayan A., Mashek D.G. (2017). Breaking fat: The Regulation and Mechanisms of Lipophagy. Biochim. Biophys. Acta Mol. Cell Biol. Lipids.

[63] Christian P., Sacco J., Adeli K. (2013). Autophagy: Emerging Roles in Lipid Homeostasis and Metabolic Control. Biochim. Biophys. Acta Mol. Cell Biol. Lipids.

[64] Busiello R.A., Savarese S., Lombardi A. (2015). Mitochondrial Uncoupling Proteins and Energy Metabolism. Front. Physiol..

[65] Chunjuan L., He X., Shi Z., Li C., Guo F., Li S., Li Y., Na L., Sun C. (2015). Ursolic Acid Increases Energy Expenditure through Enhancing Free Fatty Acid Uptake and β-oxidation via an UCP3/AMPK-dependent Pathway in Skeletal Muscle. Mol. Nutr. Food Res..

