# Supplementary material for: Comparative Analysis of Metabolite Profiling of Momordica charantia Leaf and the Anti-Obesity Effect through Regulating Lipid Metabolism
Source: Int J Environ Res Public Health. 2021 May 24;18(11):5584. doi: 10.3390/ijerph18115584 (PMC8197276; doi:10.3390/ijerph18115584)
Supplement: Supplementary file 1 [file ijerph-18-05584-s001.zip › ijerph-1216786-supplementary.pptx]

## Slide 1
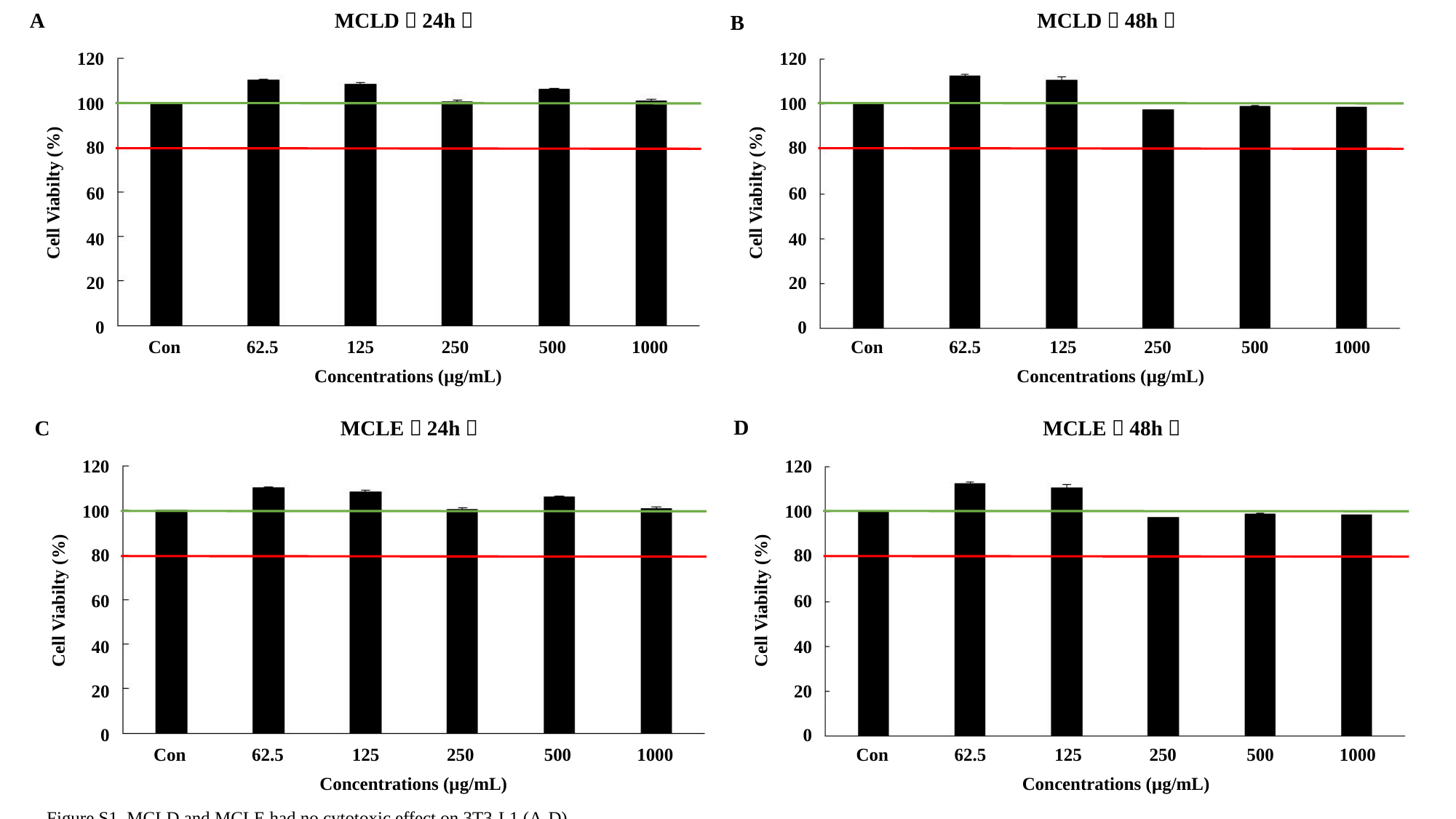

A
MCLD（24h）
Cell Viabilty (%)
120
100
80
60
40
20
0
Con
62.5
125
250
500
1000
Concentrations (μg/mL)
MCLD（48h）
Cell Viabilty (%)
120
100
80
60
40
20
0
Con
62.5
125
250
500
1000
Concentrations (μg/mL)
B
D
C
MCLE（24h）
Cell Viabilty (%)
120
100
80
60
40
20
0
Con
62.5
125
250
500
1000
Concentrations (μg/mL)
MCLE（48h）
Cell Viabilty (%)
120
100
80
60
40
20
0
Con
62.5
125
250
500
1000
Concentrations (μg/mL)
Figure S1. MCLD and MCLE had no cytotoxic effect on 3T3-L1 (A-D).

## Slide 2
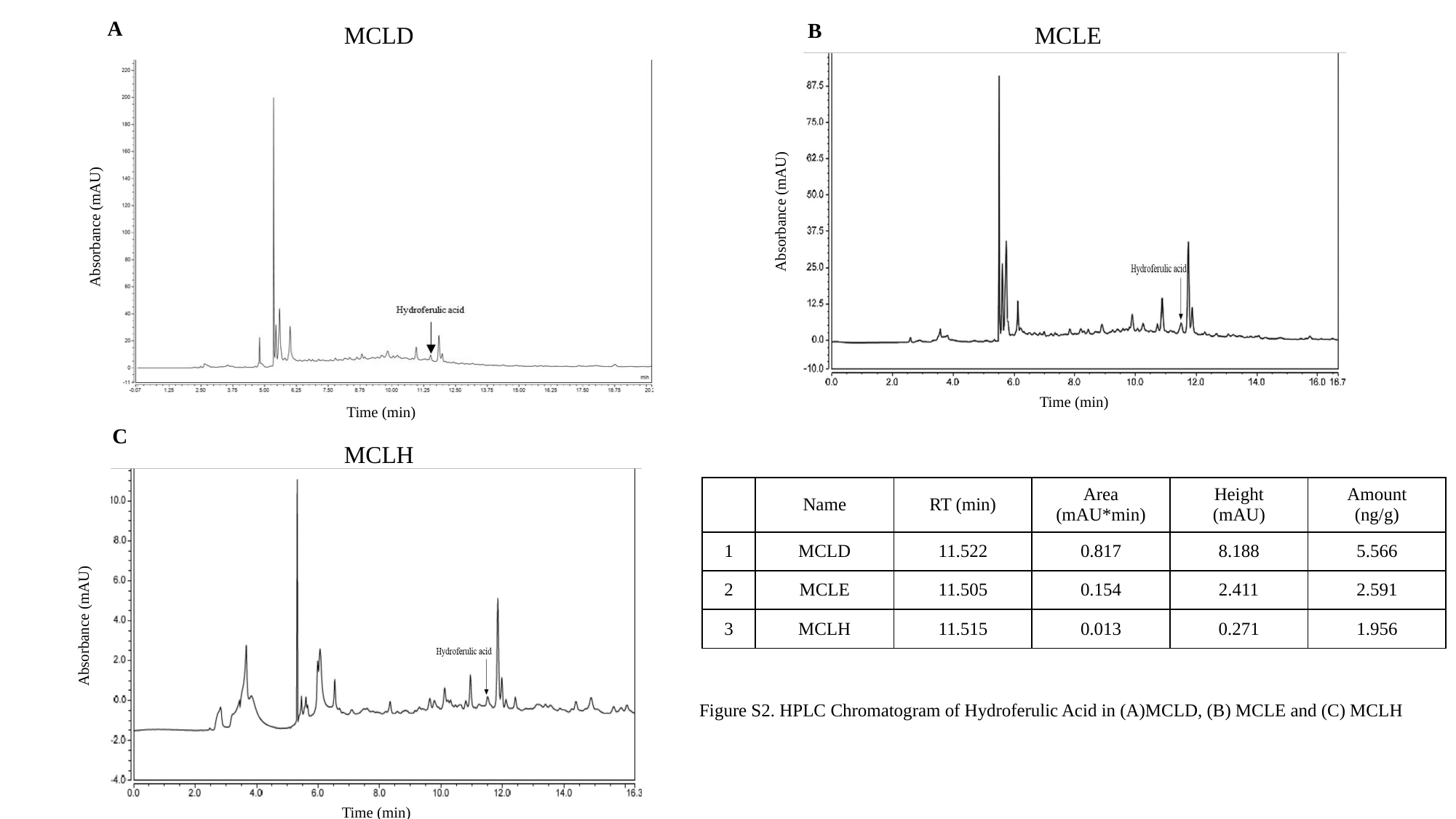

A
B
MCLD
MCLE
Absorbance (mAU)
Time (min)
Absorbance (mAU)
Time (min)
C
MCLH
Absorbance (mAU)
Time (min)
| | Name | RT (min) | Area (mAU\*min) | Height (mAU) | Amount (ng/g) |
| --- | --- | --- | --- | --- | --- |
| 1 | MCLD | 11.522 | 0.817 | 8.188 | 5.566 |
| 2 | MCLE | 11.505 | 0.154 | 2.411 | 2.591 |
| 3 | MCLH | 11.515 | 0.013 | 0.271 | 1.956 |
Figure S2. HPLC Chromatogram of Hydroferulic Acid in (A)MCLD, (B) MCLE and (C) MCLH

## Slide 3
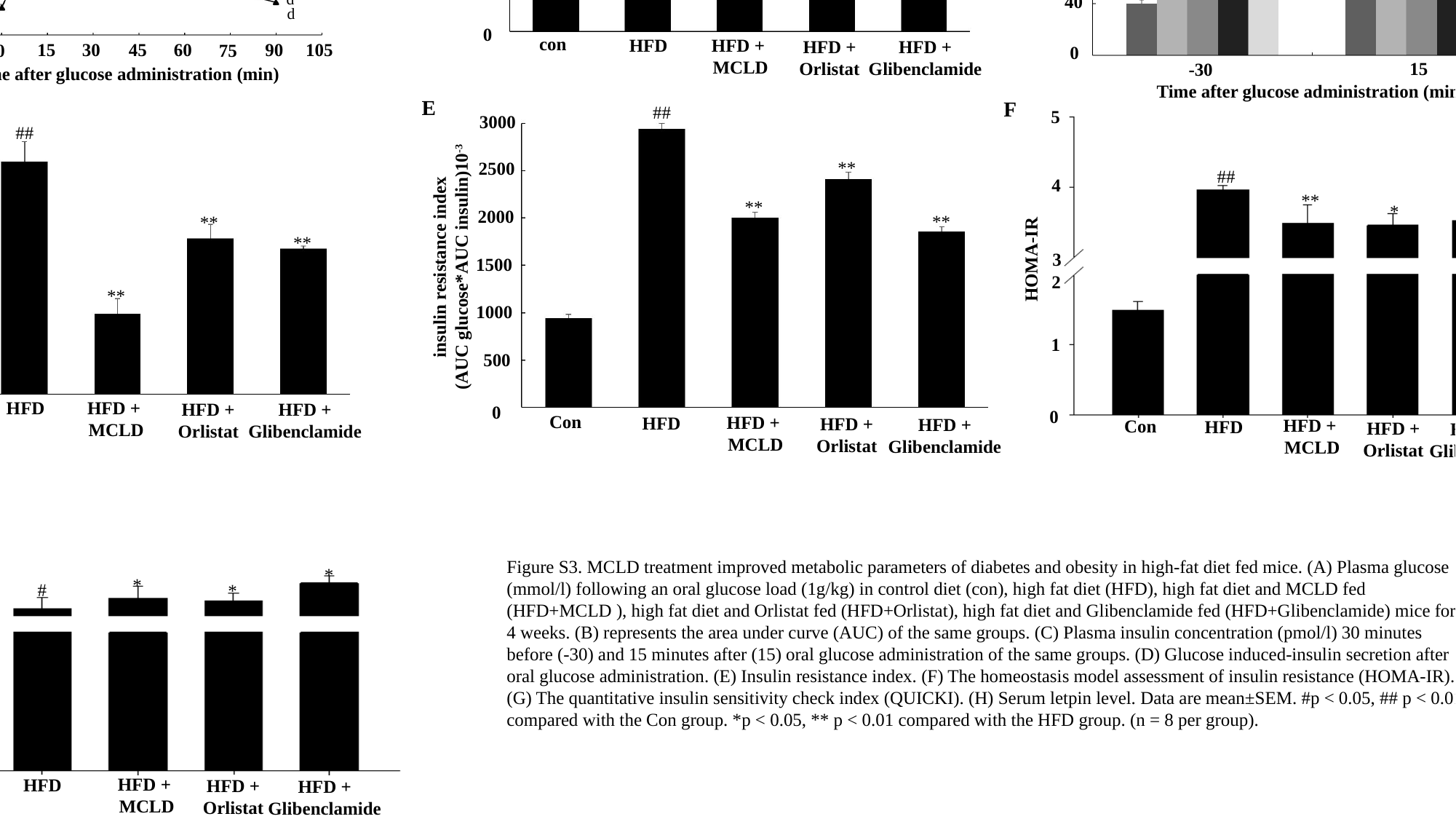

B
1800
1200
900
300
0
con
HFD
HFD +
 MCLD
HFD + Orlistat
HFD + Glibenclamide
Plasma glucose AUC
(mmol/min)
1500
600
##
*
*
**
A
25
Con
HFD
HFD + MCLD
Plasma glucose (mmol/l)
15
30
45
90
60
105
-30
-15
0
75
HFD + Orlistat
20
HFD + Glibenclamide
15
a
b
10
c
d
d
5
Time after glucose administration (min)
C
240
Con
HFD
HFD + MCLD
200
HFD + Orlistat
Plasma insulin (pmol/l)
HFD + Glibenclamide
160
120
80
40
0
15
-30
##
**
**
**
#
Time after glucose administration (min)
D
160
∆ insulin (pmol/l)
120
80
40
0
Con
HFD
HFD +
MCLD
HFD + Orlistat
HFD + Glibenclamide
##
**
**
**
E
##
3000
2000
1500
500
0
Con
HFD
HFD +
 MCLD
HFD + Orlistat
HFD + Glibenclamide
insulin resistance index
(AUC glucose*AUC insulin)10-3
2500
1000
**
**
**
F
5
##
4
*
**
*
3
HOMA-IR
2
1
0
Con
HFD
HFD + Orlistat
HFD + Glibenclamide
HFD +
 MCLD
G
0.8
0.6
*
*
*
0.5
QUICKI
0.2
##
0.1
0.0
Con
HFD
HFD + Orlistat
HFD + Glibenclamide
#
HFD +
 MCLD
Figure S3. MCLD treatment improved metabolic parameters of diabetes and obesity in high-fat diet fed mice. (A) Plasma glucose (mmol/l) following an oral glucose load (1g/kg) in control diet (con), high fat diet (HFD), high fat diet and MCLD fed (HFD+MCLD ), high fat diet and Orlistat fed (HFD+Orlistat), high fat diet and Glibenclamide fed (HFD+Glibenclamide) mice for 4 weeks. (B) represents the area under curve (AUC) of the same groups. (C) Plasma insulin concentration (pmol/l) 30 minutes before (-30) and 15 minutes after (15) oral glucose administration of the same groups. (D) Glucose induced-insulin secretion after oral glucose administration. (E) Insulin resistance index. (F) The homeostasis model assessment of insulin resistance (HOMA-IR). (G) The quantitative insulin sensitivity check index (QUICKI). (H) Serum letpin level. Data are mean±SEM. #p < 0.05, ## p < 0.01 compared with the Con group. *p < 0.05, ** p < 0.01 compared with the HFD group. (n = 8 per group).

## Slide 4
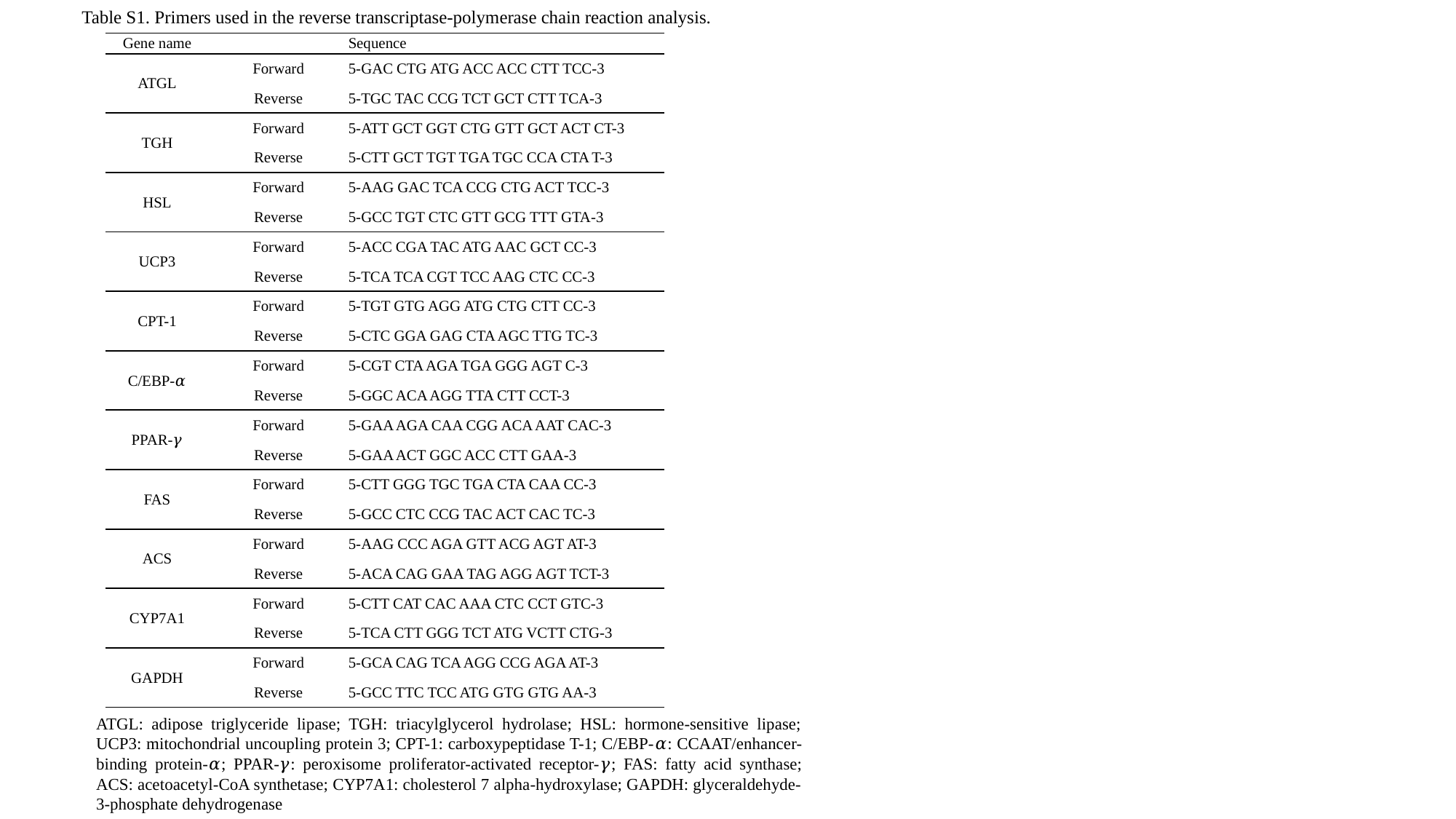

Table S1. Primers used in the reverse transcriptase-polymerase chain reaction analysis.
| Gene name | | Sequence |
| --- | --- | --- |
| ATGL | Forward | 5-GAC CTG ATG ACC ACC CTT TCC-3 |
| | Reverse | 5-TGC TAC CCG TCT GCT CTT TCA-3 |
| TGH | Forward | 5-ATT GCT GGT CTG GTT GCT ACT CT-3 |
| | Reverse | 5-CTT GCT TGT TGA TGC CCA CTA T-3 |
| HSL | Forward | 5-AAG GAC TCA CCG CTG ACT TCC-3 |
| | Reverse | 5-GCC TGT CTC GTT GCG TTT GTA-3 |
| UCP3 | Forward | 5-ACC CGA TAC ATG AAC GCT CC-3 |
| | Reverse | 5-TCA TCA CGT TCC AAG CTC CC-3 |
| CPT-1 | Forward | 5-TGT GTG AGG ATG CTG CTT CC-3 |
| | Reverse | 5-CTC GGA GAG CTA AGC TTG TC-3 |
| C/EBP-𝛼 | Forward | 5-CGT CTA AGA TGA GGG AGT C-3 |
| | Reverse | 5-GGC ACA AGG TTA CTT CCT-3 |
| PPAR-𝛾 | Forward | 5-GAA AGA CAA CGG ACA AAT CAC-3 |
| | Reverse | 5-GAA ACT GGC ACC CTT GAA-3 |
| FAS | Forward | 5-CTT GGG TGC TGA CTA CAA CC-3 |
| | Reverse | 5-GCC CTC CCG TAC ACT CAC TC-3 |
| ACS | Forward | 5-AAG CCC AGA GTT ACG AGT AT-3 |
| | Reverse | 5-ACA CAG GAA TAG AGG AGT TCT-3 |
| CYP7A1 | Forward | 5-CTT CAT CAC AAA CTC CCT GTC-3 |
| | Reverse | 5-TCA CTT GGG TCT ATG VCTT CTG-3 |
| GAPDH | Forward | 5-GCA CAG TCA AGG CCG AGA AT-3 |
| | Reverse | 5-GCC TTC TCC ATG GTG GTG AA-3 |
ATGL: adipose triglyceride lipase; TGH: triacylglycerol hydrolase; HSL: hormone-sensitive lipase; UCP3: mitochondrial uncoupling protein 3; CPT-1: carboxypeptidase T-1; C/EBP-𝛼: CCAAT/enhancer-binding protein-𝛼; PPAR-𝛾: peroxisome proliferator-activated receptor-𝛾; FAS: fatty acid synthase; ACS: acetoacetyl-CoA synthetase; CYP7A1: cholesterol 7 alpha-hydroxylase; GAPDH: glyceraldehyde-3-phosphate dehydrogenase
